# Bone Hydatid Cyst of the Hip

**DOI:** 10.4269/ajtmh.26-0171

**Published:** 2026-07-09

**Authors:** Bienvenu Jean Celien Okouango, Kamena Mwana-Yile Hassan, Mustapha Fadili

**Affiliations:** ^1^ Traumatology and Orthopaedics Department, Ibn Rochd University Hospital, HASSAN II University, Casablanca, Morocco;; ^2^ Infectious Diseases Department, Ibn Rochd University Hospital, HASSAN II University, Casablanca, Morocco

## INTRODUCTION

**Figure 1.**
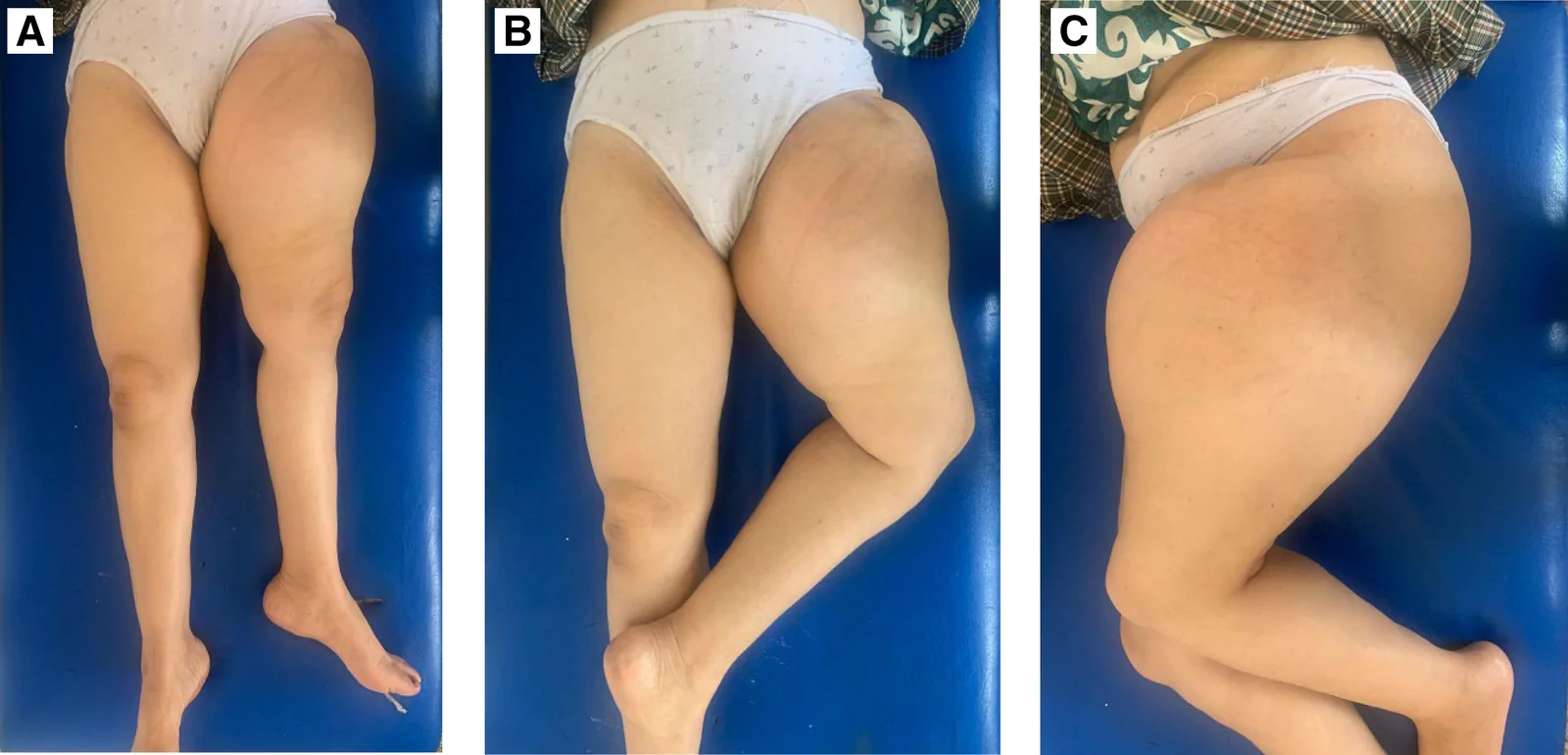
A mass is shown in the upper-left thigh area in the three panels.

A 57-year-old woman with a history of an echinococcal cyst in her left hip, treated with medical and surgical therapy 18 years ago, presented with a large mass in her left thigh. This mass had been present for 5 years. The mass extended from the groin to the proximal two-thirds of her thigh, affecting the medial, anterior, and lateral regions. It measured 33 cm along its longest axis (Figure 1). It was attached to the underlying tissues and was firm, nontender, and associated with collateral venous circulation.

**Figure 2.**
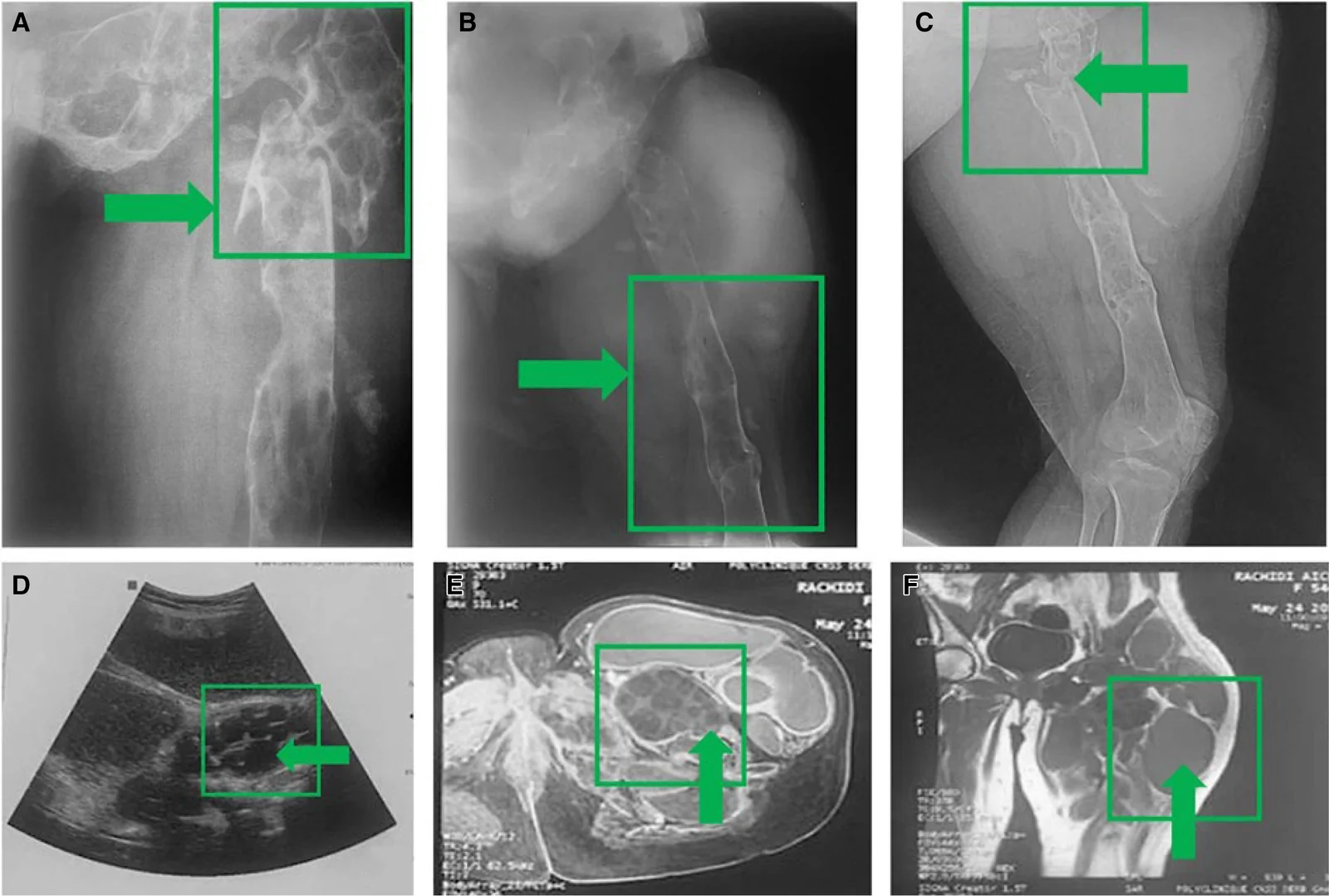
A cyst is shown in the X-rays and magnetic resonance images (MRIs) of the upper-left thigh and hip area. (**A–C**) Lytic lacunar lesions clustered to form a honeycomb pattern, with thinning and a break in the cortical bone, as well as a pathological fracture located in the subtrochanteric region. (**D**) A cyst shown in an ultrasound image displaying. (**E** and **F**) An MRI of the thigh revealed a multilocular lesion measuring 26.5 cm by 18.7 cm, along with a cystic mass at the root of the thigh.

Plain radiographs of the left hip and thigh revealed several lacunar lesions of varying sizes with poorly defined borders, along with periosteal reaction, cortical thinning, and a cortical fracture at the femoral shaft (Figure 2A–C). There was a loss of sphericity in the femoral head, a poorly defined lacunar lesion in the obturator fossa, and a disruption of continuity in the subtrochanteric region. A soft-tissue ultrasound revealed an alveolar cyst with a wall separating it from surrounding vascular structures (Figure 2D). Magnetic resonance imaging revealed a multilocular (vesicular) cyst measuring 26.5 × 18.7 cm with high signal intensity on T2 and diffuse and multilocular bone involvement in the left femur, iliac bone, and left acetabulum, visible at low intensity on T1-weighted imaging.

Hydatid serology yielded a positive result; HIV-1 and HIV-2 serology results were negative. The decision was made not to perform surgery, but to prescribe a 6-month course of albendazole. The patient died 2 years later of unrelated causes.

*Echinococcus granulosus*, a cestode, lives as an adult tapeworm in the guts of dogs. Humans acquire infection via the oral route by ingesting embryonated eggs released by canid-inhabiting tapeworms in the feces.^1^ Organ involvement in humans, most often the liver, less often the lungs, and even less often axial and other bones, reflects hematogenous dissemination.^2^ Clinical examinations are generally uninformative unless there are anaphylactoid or allergic symptoms.^3^ Bone involvement manifestations may reflect local effects, such as a limp or a palpable mass, as in the present case.^4^ The diagnosis is generally based on imaging, supplemented in unclear cases by serology. The WHO recommends albendazole for medical treatment of viable parasites.^5^
